# A Novel *COL4A4* Mutation Identified in a Chinese Family with Thin Basement Membrane Nephropathy

**DOI:** 10.1038/srep20244

**Published:** 2016-02-02

**Authors:** Yan Xu, Min Guo, Hui Dong, Wei Jiang, Ruixia Ma, Shiguo Liu, Shenqian Li

**Affiliations:** 1 Department of nephrology, the Affiliated Hospital of Qingdao University, Qingdao, 266003, China; 2 Prenatal diagnosis center, the Affiliated Hospital of Qingdao University, Qingdao, 266003, China; 3 Department of urological surgery, the Affiliated Hospital of Qingdao University, Qingdao, 266001, China

## Abstract

Thin basement membrane nephropathy (TBMN) is often attributable to mutations in the COL4A3 or *COL4A4* genes that encode the α3 and α4 chains of type IV collagen, respectively, a major structural protein in the glomerular basement membrane. The aim of this study was to explore a new disease-related genetic mutation associated with the clinical phenotype observed in a Chinese Han family with autosomal dominant TBMN. We conducted a clinical and genetic study comprising seven members of this TBMN family. Mutation screening for COL4A3 and *COL4A4* was carried out by direct sequencing. The RNA sequences associated with both proteins were also analyzed with reverse transcription PCR and TA cloning. The result showed that every affected patient had a novel heterozygous splicing mutation in *COL4A4* (c.1459 + 1G > A), which led to the elimination of the entire exon 21 from the *COL4A4* cDNA and resulted in the direct splicing of exons 20 and 22. This in turn caused a frameshift mutation after exon 20 in the open reading frame of *COL4A4*. In conclusion, we describe a novel splicing mutation in *COL4A4* that results in TBMN. This analysis increases our understanding of TBMN phenotype-genotype correlations, which should facilitate more accurate diagnosis and prenatal diagnosis of TBMN.

Thin basement membrane nephropathy (TBMN), also called benign familial hematuria, is the most common cause of inherited persistent microscopic hematuria in children and adults that occurs in at least 1% of the population^1^,^2^. It is characterized by persistent hematuria, minimal proteinuria, normal renal function and a uniformly thinned glomerular basement membrane(GBM)^2^,^3^. Although renal function of TBMN patients does not progress to chronic renal failure (CRF) or end stage renal disease (ESRD), about 10–20% patients with TBMN will development to ESRD in later life at a mean age of 60 years^4^. Currently, as no clear evidence-based treatment protocols are available for TBMN, early and accurate diagnosis for an individual patient is very important for early effective prevention of ESRD.

Autosomal dominant mutations of COL4A3 or *COL4A4* are associated with TBMN^5^. *COL4A3* and *COL4A4* encode the α3 and α4 chains of type IV collagen respectively, which are the major structural components of the GBM. The defects in *COL4A3* or *COL4A4* may lead to type IV collagen related nephropathies that comprise a spectrum of phenotypes ranging from the severe phenotype Alport syndrome (AS) to its mild variants, TBMN^6^. Since the first *COL4A4* mutation in TBMN has been described by Lemmink in 1996^7^, at present, at least twenty one different mutations have been identifiedscattered in most of the coding exons of human *COL4A3* or *COL4A4 a*nd no mutational “hotspots” were found^8^. Most mutations can result in single nucleotide substitutions which cause missense or nonsense mutations. In addition, six insertion or deletion mutations have been reported^3^. Although many different mutations in *COL4A3* and *COL4A4* have already been identified, a valid mutational spectrum of these two genes and genotype–phenotype correlation for TBMN or AS is not yet fully known. Therefore, it is essential to identify new mutations in *COL4A3* and *COL4A4* by direct sequencing to clarify their clinical phenotype and to assess the prognosis for TBMN which can help then understand this disease, and other Type IV collagen related diseases on the basis of the genotype and made further it possible to carry on prenatal diagnosis in affected members.

In this present study, we investigated a pedgree with TBMN from Shandong provience, China and performed mutational analyses in COL4A3 and *COL4A4*, to add to the existing genotype-phenotype correlations within these genes.

## Results

### Clinical phenotypeof proband

**Figure 1:**
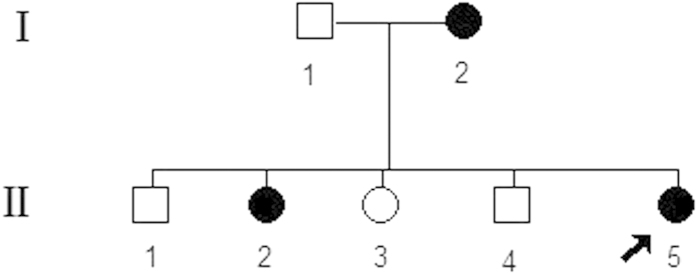
Pedigree for the family with TBMN.

The proband (II5, Fig. 1) presented with continuous microhematuria and microalbuminuria over 6 years. The pleomorphism of the erythrocytes in the urinary sediment indicated the glomerular origin of the cells. The glomerular filtration rate was normal but the urinary osmolality was slightly decreased (676 mOsm/kg). There were no signs of arterial hypertension, hearing loss, or lenticonus or macular flecks. The detailed clinical festures and urine routine test of proband and the carriers (II2 and I2, Fig. 1) were shown in Table 1.

**Figure 2:**
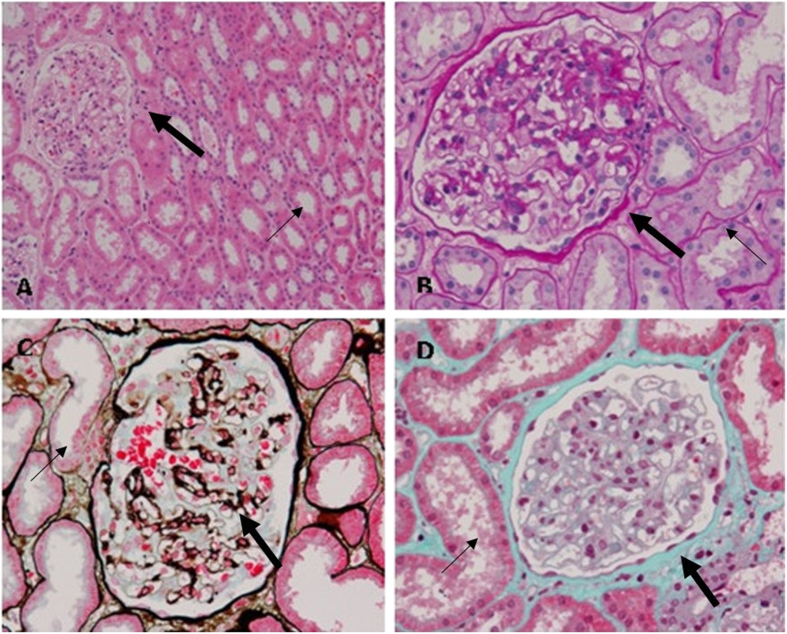
Light microscopy of renal samples in TBMN. Light microscopy of renal samples in TBMN shows almost normal glomerular histology. (**A**) PAS staining (200×) The large arrow indicates the glomerulus and the thin arrow indicated the kidney tubulus. (**B**) PAS staining (400×): The GBM and mesangial matrix were dyed fuchsia. PAS staining showed almost normal glomerular histology with only occasional mild mesangial cellular proliferation and matrix expansion. (**C**) PASM staining (400×): The GBM and mesangial matrix were dyed black and there was no obvious immune complex deposits. (**D**) Masson staining (400×): The GBM, mesangial matrix and renal interstitiumwere dyed green and interstitial fibrosis was moderate (++). No obvious immune complex deposits were observed.

**Figure 3:**
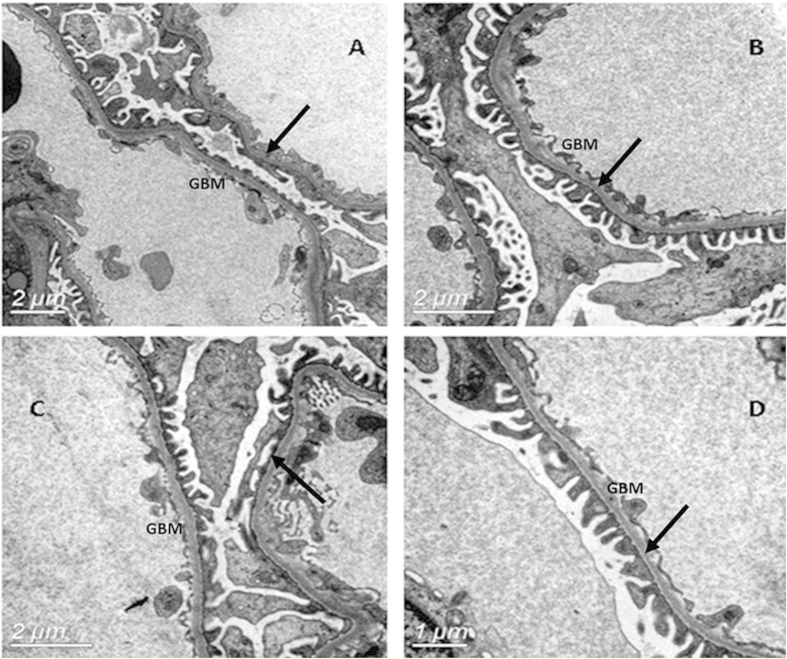
Electron microscopic of the GBM in TBMN using quick-freezing showed uniformly thinned GBM. GBM located between the fenestrated endothelial cells and the podocyte foot processes. The arrows showed the characteristically thinned GBM and few podocyte fusions. Magnifications: ×4000 in (**A–C**) and ×8000 in (**D**).

**Figure 4:**
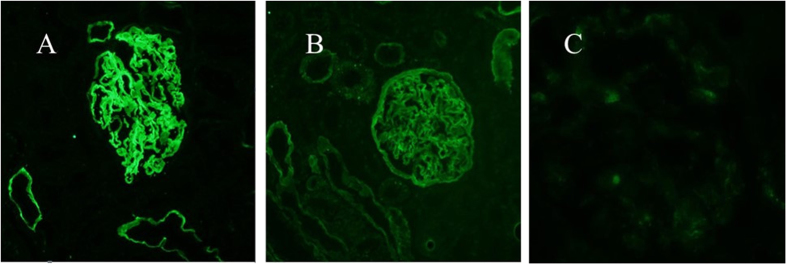
Immunofluorescence for α3 (**A**) and α5 (**B**) chain of collagen IV and IgM (**C**) in renal glomeruli from the TBMN probandII5. (**A,B**): Collagen IV immunofluorescence analysis exhibited positive collagen 3 (IV) and 5 (IV) immunostaining in kidney sections. (**C**) showed diffuse distribution of IgM, which was mainly in mesangial area with weak fluorescence intensity.

Immunohistochemical renal biopsy of the proband (Fig. 2 A,B) showed almost normal glomerular histology with only occasional mild mesangial cellular proliferation and matrix expansion under light microscopy in Periodic Acid-Schiff staining (PAS). Periodic Acid-Silver Methenamine (PASM)can dye GBM and mesangial matrix black and Masson staining can dye the GBM, mesangial matrix as well as renal interstitium green. The result of PASM and Masson staining indicated no obvious immune complex deposits in kidney (Fig. 2 C,D). The percentage of focal glomerular sclerosis was approximately 7.3% and interstitial fibrosis was moderate (++). A few erythrocyte casts and protein casts could be identified in the tube, while the interstitial capillaries were normal. Ultrastructural images (Fig. 3) showed characteristically thinned GBM of 144–204 nm, and few podocyte fusions were found. No other structural abnormalities were apparent. There was no mesangial matrix expansion and no dense materials were visible in the mesangial area or in the basement membrane. Immunofluorescence for IgM showed diffuse distribution in mesangial area with weak fluorescence intensity (Fig. 4). To further distinguish TBMN from early stage AS, immunofluorescence for type IV collagen α3 and α5 chains were performed. Immunofluorescence evaluation of the type IV collagen α3 and α5 chains in renal biopsy displayed a normal state (Fig.4). Next we carried on genetic testing directly toidentify the mode of inheritance and the nature of the underlying mutation.

### Mutation screening of COL4A3 and COL4A4

**Figure 5:**
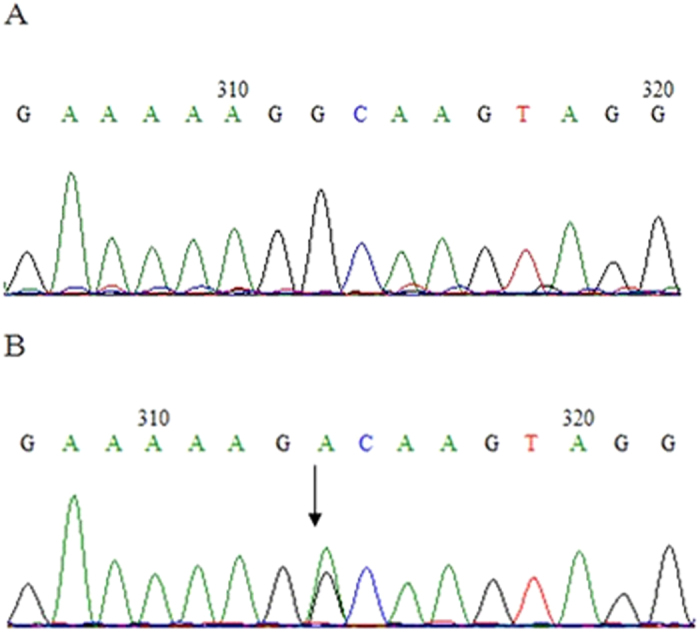
Partial nucleotide sequence showing the splice site mutation between exon 21 and 22 of *COL4A4*. (**A**) Normal DNA sequence. (**B**) Sequence of DNA from patients, who were heterozygous for the G to A mutation. The arrows indicated the position of the G to A substitution.

Analysis of the COL4A3 and COL4A4 genes of the proband(II5) revealed a heterozygous single-base alteration (G > A) that led to a substitution of guanine for adenine at position 1459 on the splice site between exon 21 and 22 of COL4A4 (c.1459 + G > A), while no mutations were found in COL4A3. This mutation was subsequently screened for across all family members. The same mutation was found only in the eldest sister (II2) and the mother (I2). Direct sequence analysis of 200 normal chromosomes from healthy control subjects of the same ethnic origin did not identify the same mutation. Therefore, this novel splice mutation is most likely the causal mutation responsible forthe TBMN phenotype in this family (Fig. 5).

### RNA analysis

**Figure 6:**
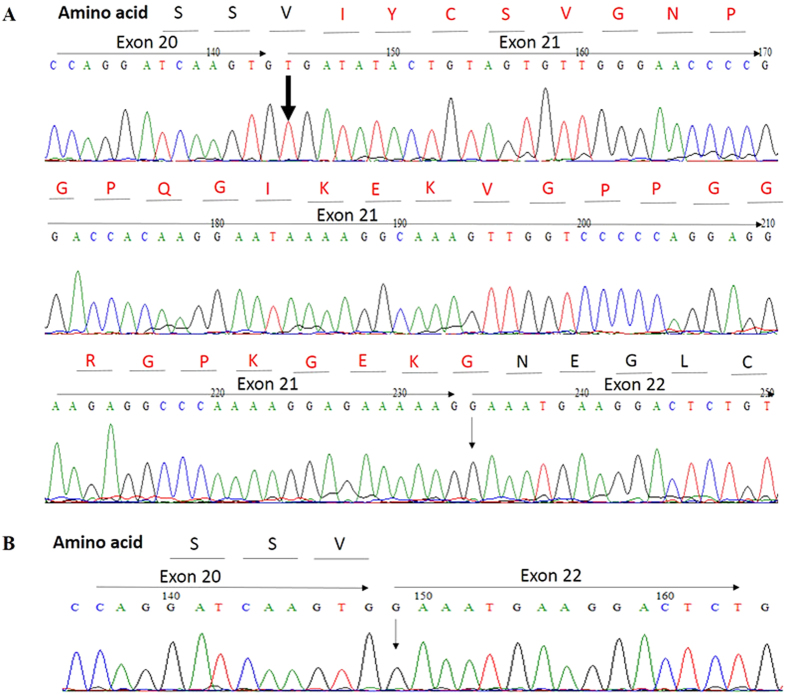
Partial RNA sequence of *COL4A4* inthe patients with TBMN and normal person. (**A**) the wild type. (**B**) the patient. The large arrow indicated the first base of exon 21 of *COL4A4*. Thin arrow indicated the first base of exon 22 of *COL4A4*.

RNA sequence analysis of the proband confirmed that the c.1459 + G > A splice mutationof COL4A4 eliminated the entire exon 21 from the *COL4A4* cDNA, with the result that exon 20 and 22 were directly spliced together (Fig. 6), which further caused a frameshift mutation after exon 20 in the coding sequence ofCOL4A4. We observed this change at the RNA level in all affected family members including II2 and I2, but not in the healthy family members.

## Discussion

The GBM is formed by a type IV collagen network. The triple-helical type IV collagen molecules form a network by associating with each other at their ends that forms the structural skeleton of the basement membranes^9^. Up till now, six different type IV collagen chains α1 to α6 have been identified, which are encoded by *COL4A1* to *COL4A6*, respectively. With the development of kidney, the α1α2 protomer is gradually replaced by the α3α4α5 protomer therefore the α3:α4:α5 chain is a major component of the GBM after birth, which is more cross-linked and resistant to degradation^10^.

Co-located on 2q36.3, *COL4A3* contains 52 exons and encodes the α3 chain (1670 amino acids) while *COL4A4* contains 48 exons and encodes the α4 chain (1690 amino acids)^11^. The α3 and α4 chains combine with α5 chains to make a complete type IV collagen molecule, which attachs to each other to form complex protein networks. Possible effects of *COLA43* and *COL4A4* mutations in TBMN could be a hamper in developmental switch to the α3α4α5 network, a decreased content of type 4 collagen chains, a reduced cross linking in the type IV collagen network and eventually adecreased thickness and stability of the GBM, resulting in TBMN or AS.

The diagnosis of TBMN is made clinically when there is persistent glomerular hematuria, minimal proteinuria (500 mg/d), normal renal function(mostly), and no other obvious extrarenal abnormalities^12^. About 40% families with TBMN have hematuria that segregates with the mutations in *COL4A3/COL4A4* locus and about two thirds of individuals with TBMN have at least one other family member with hematuria in autosomal dominant inheritance pattern^13^. Families with TBMN in whose hematuria does not segregate with the *COL4A3/COL4A4* locus can be explained by de novo mutations, incomplete penetrance of hematuria, coincidental hematuria in family members without *COL4A3 or COL4A4* mutations, or by an as yet unidentified causative gene locus for TBMN^1^.

Several typical features of AS include haematuria, proteinuria, progressive renal failure, progressive sensorineural hearing loss(SNHL), anterior lenticonus, positive family history for haematuria, splitting and thickening of the glomerular basement membrane, and renal disease progresses from microscopic hematuria to proteinuria, progressive renal insufficiency and end-stage renal disease (ESRD)^14^. X-linked dominant AS is the major form (85% of the cases) and is associated with mutations in the X-linked *COL4A5*^15^. 15% of the cases follow autosomal-recessive inheritance (ARAS) which is caused by homozygous or compound heterozygous mutations in *COL4A3* and *COL4A4*^16^. The remaining 5% autosomal-dominant type of AS (ADAS) were due to heterozygous *COL4A3* or *COL4A4*mutation^17^.

TBMN and AS have morbigenous mutations in the same genes (*COL4A3* or *COL4A4*). Lemmink *et al.* first suggested patients with TBMN that is linked to chromosome 2 are heterozygous for mutations in either *COL4A3* or *COL4A4*, and they represent a carrier status for ARAS^7^. To date, six mutations have been described that are common to both conditions (G464V, G1015E in *COL4A3* and R1377X, S969X, a184-bp deletion in intron 24 and exon 25, and an 18-bp deletion in exon 25 in *COL4A4*)^5^,^18^,^19^. These observations confirmed that TBMN represented the carrier state for ARAS at least in some families. So, it posed a challenge to accurately diagnose the two diseases in this condition. Apart from the differences in clinical manifestations, renal biopsy can be a distinction standard. It has been reported that the mean GBM thickness in female adults was found to be 320 ± 50 nm^20^ and the World Health Organization (WHO) had proposed a threshold of 250 nm for adults in TBMN. While in AS, renal biopsies are characterized by irregular thickening of the GBM^21^. More precisely distinction may refer to specific disease-causing genes.

To date, about 21 mutations in *COL4A3* and *COL4A4* have been idenfied in TBMN, and most of these are single nucleotide substitutions that are different in each family. For example, missense mutations (G957R, G960R, S969X in *COL4A4* and G464V, G532C, G584C, G596R, G695R, G985V, G1015E in *COL4A3*) all affect a glycine residue^5^,^22^,^23^,^24^,^25^, which is the only amino acid small enough to fit the triple helix structure of the collagen network. The splice site (c.1935 del18) mutation eliminates18-bp in exon 25 in *COL4A4*, producing a shift in the reading frame that leads to a truncated protein missing half of the collagenous domain^19^. IVS23–1G > C in exon 24 of *COL4A4* and IVS40-7 C > G and IVS351 G > A in *COL4A3* produce an aberrant splicing to be associated with TBMN^22^. Therefore, the identification of novel mutations in these genes is particularly important to enable diagnostic laboratories to distinguish mutations from uncommon normal variants so as to ascertain the relationship of genotype-phenotype in TBMN and AS.

In this present study, the COL4A4 c1459 + G > A was first reported in the literature as a heterozygous change in a 35-year old female proband with history of microhematuria, mild proteinuria and uniformly thinned GBM (144–204 nm). The proband did not have sensorineural hearing loss or ocular features, and her mother and sister, who was also heterozygous for this mutation, had history significant for microhematuria. The mode of inheritance was therefore suggested to be autosomal dominant. In X-linked AS, the collagen IV α5 chain, as well as the α3 and α4 chains are often absent from the GBM; and in ARAS, the α3α4α5 network is absent from the GBM^26^. Based on the above evidences we excluded X-linked dominant AS and ARAS clinically because the proband lacked the typical characteristics of AS, with normal type IV collagen α3/α5 chains and autosomal dominant inheritance pattern. ADAS and TBMN both exhibit normal GBM staining for the collagen α3(IV) chain and α5(IV) chain. ESRD and SNHL are relatively late in onset and ocular involvement is rare in ADAS. But in ADAS, the lamina densa appears to be split into multiple interlacing strands of electron-dense material; the lacunae between these strands are frequently occupied by round, electron-dense bodies; the glomerular capillary wall is diffusely thickened^14^. Therefore, we can conclude that the c1459 + G > A mutation can cause the mild phenotype TBMN rather than AS in this China Han family. If the COL4A4 c1459 + G > A also exists in ADAS patients or if it is common to both conditions still remains to be further explored.

Further investigation demonstrated the underlying pathogenic mechanism that this splice site mutation can eliminate exon 21 from the cDNA to further cause frameshift mutation after exon 20 in the open reading frame of *COL4A4* and result in changes in amino acid sequence of α4 chain. As the linear amino acid sequence of a protein is the primary structure of protein and determines its secondary, tertiary and quaternary structure, we may come to a conlusion that the COL4A4 c1459 + G > A mutation generated abnormal α4 chain of type IV collagen, then influenced the α3α4α5 protomer to form correctly, eventually affect the α3α4α5 network and structural skeleton of the sement membranes, leading to TBMN in this family.

In conclusion, we identified a novel splice pathogenic mutation that is responsible for this TBMN pedigree through aberrant splicing and frameshift mutation. To our knowledge, this is the first report of *COL4A4*mutation in any population. Our findings also demonstrate genetic heterogeneity of the mutation and add to knowledge of the correlations of phenotype–genotype in TBMN. Such knowledge might assist in understanding the mechanism of the pathophysiology of TBMN, and form the basis of more accurate and rapid diagnosis of this hereditary birth defect.

## Materials and Methods

### Patients

The subjects were a Chinese family of seven from Shandong province, China. The proband (II5), a 35-year-old woman, under wentrenal biopsy, which revealed uniformly thinned GBM. She subsequently asked for genetic counselling and prenatal diagnosis. Her mother and eldest sister were both diagnosed with having microhematuria for more than 10 years, but were negative for renal failure and hearing loss. Herfather and other three siblings, two males and onefemale, did not show any significant symptoms or abnormalities on testing (Fig. 1). After written informed consent was obtained, blood samples were collected from all family members. The study was approved by the Ethics Committee of the Affiliated Hospital of Qingdao University and the methods were carried out in accordancewith the approved guidelines.

### Mutation screening

Genomic DNA from all participating family members was extractedfrom the peripheral blood leukocytes using the phenol-chloroform method. All coding exons and splice sites of COL4A3 (NM_000091.4; 52 exons) and COL4A4(NM_000092.4; 48 exons) were PCR-amplified using gene-specific primer pairs designed with Primer5 (Premier Biosoft International, version 5.0). The annealing temperatures used were between 50–60 °C. Amplified PCR products were purified and sequenced using the appropriate PCR primers and the Big Dye Terminator Cycle Sequencing kit (Applied Biosystems, Foster City, CA, USA), and run on an automatedsequencer, ABI 3730xl (Applied Biosystems), to perform mutational analysis. DNA sequences were analyzed using the BioEdit (Borland, V7.0.1) program and compared to a reference sequence from the GenBank database.

### RNA analysis and TA cloning

RNA from 1 mL peripheral blood leucocytes was isolated with the QIA amp RNA Blood Mini kit (Qiagen, Hilden, Germany). cDNA was prepared from the total RNA with PrimeScript RT Enzyme Mix using the PrimeScript RT Reagent Kit with RNA Eraser (TaKaRa, Japan) according to the manufacturer’s instructions. RNA sequences containing the mutation site was amplified by PCR. The primers used for PCR analysis were 5′-CCTGTGCAGGCATGATAGGA-3′ and 5′-TGCCCTTTAACTCTTGATACAACCA-3′. PCR products were separated by 1% agarose gel electrophoresisand target DNA was extracted with a Gene JET Gel Extraction Kit (Thermo Scientific, Waltham, MA, USA). pMD19-T vector(1 μL; TaKaRa) was mixed and incubated with 4 μL target COL4A4 DNA at 16 °C for 30 min. This mixture was added to 100 μL TOP10 competent cells and incubated for 30 min on ice, 1 min at 42 °C, then 2 min on ice as per manufacturer’s instructions, toallow the transformation of TOP10 competent cells with the pMD19-T vector containingthe target DNA. Luria Broth (LB) was added to each tube (900 μL) and the cells were cultured at 37 °C, 150 rpm for 45 min. Then, 100 μL of the transformed cells were spread evenly on LB-agar plates containing 100 mg/mL penbritin, 24 mg/mL IPTG, and 20 mg/mL X-Gal, and cultured at 37 °C for 16h. Cells transformed with vectors containing recombinant DNA produced white colonies that were selected for further culturing at 37 °C, 250 rpm for 12 h. The plasmid DNAwas then extracted and sequencing was performed with a universal primer.

## Additional Information

**How to cite this article**: Xu, Y. *et al.* A Novel *COL4A4* Mutation Identified in a Chinese Family with Thin Basement Membrane Nephropathy. *Sci. Rep.* **6**, 20244; doi: 10.1038/srep20244 (2016).

## Figures and Tables

**Table 1:** Clinical festures and urine routine test of proband and carriers.

items	II5	II2	I2	normal range
age (year)	35	42	69	—
SP (mmHg)	131	128	140	≤140
DP (mmHg)	70	80	75	≤90
BMI (Kg/m2)	22.38	20.8	23.4	18.5~23.9
URBC (/mL)	7.2 × 10^6^	5.4 × 10^6^	9.6 × 10^6^	0.8~1
NAG (u/g.cr)	6.4	7.9	9.5	≤16.5
C3 (mg/L)	2	1.2	2.3	≤2.76
Uosm (mOsm/kg)	676	727	580	>800
Upr (g/24 h)	0.61	0.43	0.58	≤0.4
α3-chain	normal	—	—	normal
α5-chain	normal	—	—	normal

URBC: urinary red blood cell Upr: urine protein.

Uosm: urinary osmolality.

## References

[b1] Savige J. *et al.* Thin basement membrane nephropathy. Kidney Int 64, 1169–1178, 10.1046/j.1523-1755.2003.00234.x (2003). 12969134

[b2] Gregory M. C. The clinical features of thin basement membrane nephropathy. Semin Nephrol 25, 140–145 (2005). 15880323 10.1016/j.semnephrol.2005.01.004

[b3] Tryggvason K. & Patrakka J. Thin basement membrane nephropathy. J Am Soc Nephrol 17, 813–822, 10.1681/ASN.2005070737 (2006). 16467446

[b4] Pierides A. *et al.* Clinico-pathological correlations in 127 patients in 11 large pedigrees, segregating one of three heterozygous mutations in the COL4A3/ COL4A4 genes associated with familial haematuria and significant late progression to proteinuria and chronic kidney disease from focal segmental glomerulosclerosis. Nephrol Dial Transplant 24, 2721–2729, 10.1093/ndt/gfp158 (2009). 19357112

[b5] Badenas C. *et al.* Mutations in the COL4A4 and COL4A3 genes cause familial benign hematuria. J Am Soc Nephrol 13, 1248–1254 (2002). 11961012 10.1681/ASN.V1351248

[b6] Deltas C., Pierides A. & Voskarides K. Molecular genetics of familial hematuric diseases. Nephrol Dial Transplant 28, 2946–2960, 10.1093/ndt/gft253 (2013). 24046192

[b7] Lemmink H. H. *et al.* Benign familial hematuria due to mutation of the type IV collagen alpha4 gene. J Clin Invest 98, 1114–1118, 10.1172/jci118893 (1996). 8787673 PMC507532

[b8] Rana K. *et al.* The genetics of thin basement membrane nephropathy. Semin Nephrol 25, 163–170 (2005). 15880327 10.1016/j.semnephrol.2005.01.008

[b9] Hudson B. G., Tryggvason K., Sundaramoorthy M. & Neilson E. G. Alport’s syndrome, Goodpasture’s syndrome, and type IV collagen. N Engl J Med 348, 2543–2556, 10.1056/NEJMra022296 (2003). 12815141

[b10] Miner J. H. & Sanes J. R. Collagen IV alpha 3, alpha 4, and alpha 5 chains in rodent basal laminae: sequence, distribution, association with laminins, and developmental switches. J Cell Biol 127, 879–891 (1994). 7962065 10.1083/jcb.127.3.879 PMC2120241

[b11] Mariyama M., Zheng K., Yang-Feng T. L. & Reeders S. T. Colocalization of the genes for the alpha 3(IV) and alpha 4(IV) chains of type IV collagen to chromosome 2 bands q35-q37. Genomics 13, 809–813 (1992). 1639407 10.1016/0888-7543(92)90157-n

[b12] Perry G. J. *et al.* Thin-membrane nephropathy–a common cause of glomerular haematuria. Med J Aust 151, 638–642 (1989). 2593909 10.5694/j.1326-5377.1989.tb139637.x

[b13] Buzza M., Wilson D. & Savige J. Segregation of hematuria in thin basement membrane disease with haplotypes at the loci for Alport syndrome. Kidney Int 59, 1670–1676, 10.1046/j.1523-1755.2001.0590051670.x (2001). 11318937

[b14] Kashtan C. E. In GeneReviews(R) (eds Pagon R. A. *et al.* ) (University of Washington, Seattle University of Washington, Seattle. All rights reserved., 1993).

[b15] Barker D. F. *et al.* Identification of mutations in the COL4A5 collagen gene in Alport syndrome. Science 248, 1224–1227 (1990). 2349482 10.1126/science.2349482

[b16] Lemmink H. H. *et al.* Mutations in the type IV collagen alpha 3 (COL4A3) gene in autosomal recessive Alport syndrome. Hum Mol Genet 3, 1269–1273 (1994). 7987301 10.1093/hmg/3.8.1269

[b17] Jefferson J. A. *et al.* Autosomal dominant Alport syndrome linked to the type IV collage alpha 3 and alpha 4 genes (COL4A3 and COL4A4). Nephrol Dial Transplant 12, 1595–1599 (1997). 9269635 10.1093/ndt/12.8.1595

[b18] Buzza M. *et al.* COL4A4 mutation in thin basement membrane disease previously described in Alport syndrome. Kidney Int 60, 480–483, 10.1046/j.1523-1755.2001.060002480.x (2001). 11473630

[b19] Gross O., Netzer K. O., Lambrecht R., Seibold S. & Weber M. Novel COL4A4 splice defect and in-frame deletion in a large consanguine family as a genetic link between benign familial haematuria and autosomal Alport syndrome. Nephrol Dial Transplant 18, 1122–1127 (2003). 12748344 10.1093/ndt/gfg157

[b20] Steffes M. W. *et al.* Quantitative glomerular morphology of the normal human kidney. Lab Invest 49, 82–86 (1983). 6865334

[b21] Spear G. S. & Slusser R. J. Alport’s syndrome. Emphasizing electron microscopic studies of the glomerulus. Am J Pathol 69, 213–224 (1972). 4343992 PMC2032644

[b22] Wang Y. Y. *et al.* COL4A3 mutations and their clinical consequences in thin basement membrane nephropathy (TBMN). Kidney Int 65, 786–790, 10.1111/j.1523-1755.2004.00453.x (2004). 14871398

[b23] Tazon Vega B. *et al.* Autosomal recessive Alport’s syndrome and benign familial hematuria are collagen type IV diseases. Am J Kidney Dis 42, 952–959 (2003). 14582039 10.1016/j.ajkd.2003.08.002

[b24] Buzza M. *et al.* Mutations in the COL4A4 gene in thin basement membrane disease. Kidney Int 63, 447–453, 10.1046/j.1523-1755.2003.00780.x (2003). 12631110

[b25] Ozen S. *et al.* Benign familial hematuria associated with a novel COL4A4 mutation. Pediatr Nephrol 16, 874–877 (2001). 11685592 10.1007/s004670100673

[b26] Savige J. Alport syndrome: its effects on the glomerular filtration barrier and implications for future treatment. J Physiol 592, 4013–4023, 10.1113/jphysiol.2014.274449 (2014). 25107927 PMC4198011

